# Residues R1075, D1090, R1095, and C1130 Are Critical in ADAMTS13 TSP8-Spacer Interaction Predicted by Molecular Dynamics Simulation

**DOI:** 10.3390/molecules26247525

**Published:** 2021-12-12

**Authors:** Zhiwei Wu, Junxian Yang, Xubin Xie, Guangjian Liu, Ying Fang, Jianhua Wu, Jiangguo Lin

**Affiliations:** 1 Institute of Biomechanics/School of Biology and Biological Engineering, South China University of Technology, Guangzhou 510006, China; 201820136157@mail.scut.edu.cn (Z.W.); 201820136164@mail.scut.edu.cn (J.Y.); bixiexubin91@mail.scut.edu.cn (X.X.); yfang@scut.edu.cn (Y.F.); wujianhua@scut.edu.cn (J.W.); 2 Research Department of Medical Sciences, Guangdong Provincial People’s Hospital, Guangdong Academy of Medical Sciences, Guangzhou 510080, China; liuguangjian@gdph.org.cn; 3 Department of Emergency Medicine, Guangdong Provincial People’s Hospital, Guangdong Academy of Medical Sciences, Guangzhou 510080, China

**Keywords:** ADAMTS13, VWF, thrombotic thrombocytopenic purpura, auto-inhibition, molecular dynamics simulation

## Abstract

ADAMTS13 (A Disintegrin and Metalloprotease with Thrombospondin type 1 repeats, member 13) cleaves von Willebrand Factor (VWF) multimers to limit the prothrombotic function of VWF. The deficiency of ADAMTS13 causes a lethal thrombotic microvascular disease, thrombotic thrombocytopenic purpura (TTP). ADAMTS13 circulates in a “closed” conformation with the distal domain associating the Spacer domain to avoid off-target proteolysis or recognition by auto-antibodies. However, the interactions of the distal TSP8 domain and the Spacer domain remain elusive. Here, we constructed the TSP8-Spacer complex by a combination of homology modelling and flexible docking. Molecular dynamics simulation was applied to map the binding sites on the TSP8 or Spacer domain. The results predicted that R1075, D1090, R1095, and C1130 on the TSP8 domain were key residues that interacted with the Spacer domain. R1075 and R1095 bound exosite-4 tightly, D1090 formed multiple hydrogen bonds and salt bridges with exosite-3, and C1130 interacted with both exosite-3 and exosite-4. Specific mutations of exosite-3 (R568K/F592Y/R660K/Y661F/Y665F) or the four key residues (R1075A/D1090A/R1095A/C1130A) impaired the binding of the TSP8 domain to the Spacer domain. These results shed new light on the understanding of the auto-inhibition of ADAMTS13.

## 1. Introduction

ADAMTS13 is a metalloprotease that comprises a metalloproteinase (MP), a disintegrin-like (Dis), a thrombospondin-1 type 1 repeat (TSP1), a cysteine-rich region (Cys), a Spacer, seven additional type 1 repeats (TSP2–TSP8), and two CUB (complement components C1r/C1s, Uegf and bone morphogenic protein 1) domains [1]. This protease cleaves at the Tyr1605-Met1606 scissile bond on the VWF A2 domain to prevent microvascular thrombosis [2,3]. The deficiency of ADAMTS13, induced by mutations of *ADAMTS13* or auto-antibodies against ADAMTS13, impairs its proteolytic activity and eventually leads to TTP [4].

It is believed that wild-type (WT) ADAMTS13 circulates in a “closed” conformation in which the distal TSP8-CUB2 domains interact with the Spacer domain, resulting in impaired proteolytic activity [5,6,7]. This auto-inhibition of ADAMTS13 could be relieved by the binding of the VWF D4-CK region to the C-terminal regions of ADAMTS13, which also is the first step in the interactions of ADAMTS13 with VWF [8]. This engagement facilitates the exosites on the Spacer, Cys, and Dis domains to interact with the discrete binding sites on the unfolded VWF A2 domain. The MP domain is then allosterically activated and cleaves the VWF scissile bond [9,10]. This substrate-mediated allosteric activation prevents ADAMT13 from off-target proteolysis [11] or recognition by auto-antibodies [12].

The Spacer domain is a key player in mediating the auto-inhibition of ADAMTS13. Exosite-3 (R568/F592/R660/Y661/Y665, [RFRYY]), the key epitope recognized by the auto-antibodies produced by TTP patients, is located on the Spacer domain [12,13,14] and pivotal in maintaining the “closed” conformation [5,8]. When exosite-3 undergoes a particular mutation (KYKFF), the conformation of ADAMTS13 shifts to an “open” state with increased cleavage activity of ~2.5-fold compared to WT ADAMTS13. In addition, unlike WT ADAMTS13, this gain-of-function (GoF) variant is resistant to ADAMTS13 auto-antibodies.

In 2017, Kieron South and co-workers demonstrated that although both CUB1 and CUB2 domains bound to exosite-3, only the former was capable of inhibiting the proteolytic activity of MDTCS, a proximal truncated construct of ADAMTS13 with high activity [6]. Recently, the crystal structure of the CUB1–2 domains has been resolved [15]. Arg1326, Glu1387, Glu1389, Trp1245, Trp1250, Lys1252, and Arg1272 in CUB1–2 could bind to the Spacer domain, mediating the “closed” conformation of ADAMTS13. We previously illustrated that the CUB1 domain simultaneously bound to three distinct regions of the Spacer domain by molecular dynamics (MD) simulation [16]. Increasing evidence indicates that the TSP8 domain is involved in the auto-inhibition of ADAMTS13 as well [17]. The removal of the TSP8 domain partially relieves the auto-inhibition of ADAMTS13 [7]. In addition, monoclonal antibodies, which are produced by patients with acquired TTP and recognize the TSP8 domain, increase the enzymatic cleavage activity of ADAMTS13 by ~2-fold [8]. However, the crystal structure of the TSP8 domain is still lacking. The molecular structure basis of the interaction of TSP8-Spacer remains elusive.

To characterize the interaction of TSP8-Spacer and predict the key residues on the TSP8 domain, we used computational approaches to construct the TSP8-Spacer complex and analyzed the interaction of the TSP8 domain and the Spacer domain at the atomic level with MD simulation. Arg1075, Asp1090, Arg1095, and Cys1130 on the TSP8 domain were predicted to be critical in stabilizing the TSP8-Spacer complex. GoF mutation on exosite-3 weakened the interactions of Asp1090 with exosite-3, but enhanced the bindings of Arg1075, Arg1095, and Cys1130 to exosite-4. Mutation of these four predicted residues on the TSP8 domain to Ala impaired the binding of the TSP8 domain to the Spacer domain.

## 2. Results

### 2.1. The Optimal TSP8-Spacer Docking Complex

**Figure 1:**
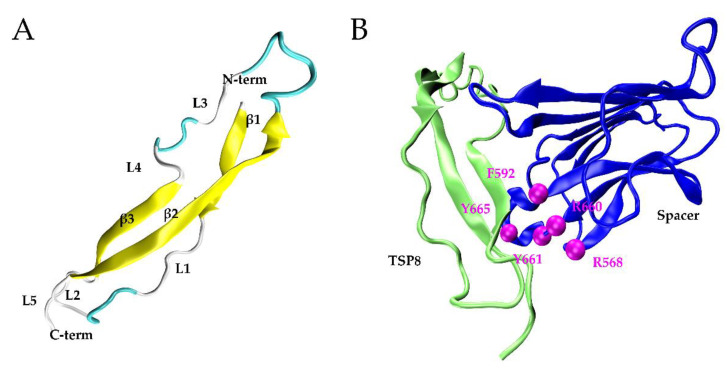
The models of the TSP8 domain and the TSP8-Spacer complex. (**A**) The best model of TSP8 domain from the Phyre2, including three β-strands (yellow), three β-turns (cyan), and five loops (grey); (**B**) conformation of the TSP8 domain (left, green) interacting with the Spacer domain (right, blue) (PDB ID: 3GHM) in Model A. Exosite-3 (magenta) of the Spacer domain was illustrated.

Ninety-two percent of TSP8 residues were modeled with 99% confidence with its template, thrombospondin-1 TSR domains 2 and 3 (PDB: 3R6B). Figure 1A showed the 3D structure of the TSP8 domain: one long β-strand (β2) anti-paralleled to two short β-strands (β1 and β3), which were connected by three turns and five loops.

After docking by SwarmDock Server, 127 TSP8-Spacer complexes were obtained. According to the two criteria (Methods), two potential TSP8-Spacer complexes, 67d and 47d, were selected. The democratic scoring [18] displayed that 67d ranked first and 47d ranked fourth. Based on this, 67d was designated as Model A and 47d was designated as Model B. Table 1 showed that more hydrogen bonds (H-bonds) and salt bridges were formed on the interface of Model A (Figure 1B), indicating it was more stable. Seven H-bonds and three salt bridges were located on the interface of Model A, involving three residues of exosite-3 (Arg568, Arg660, and Tyr665). Model B contained five H-bonds, four of which were formed by the residues of exosite-3 (Arg568, Phe592, and Arg660). No salt bridge was observed in Model B.

**Figure 2:**
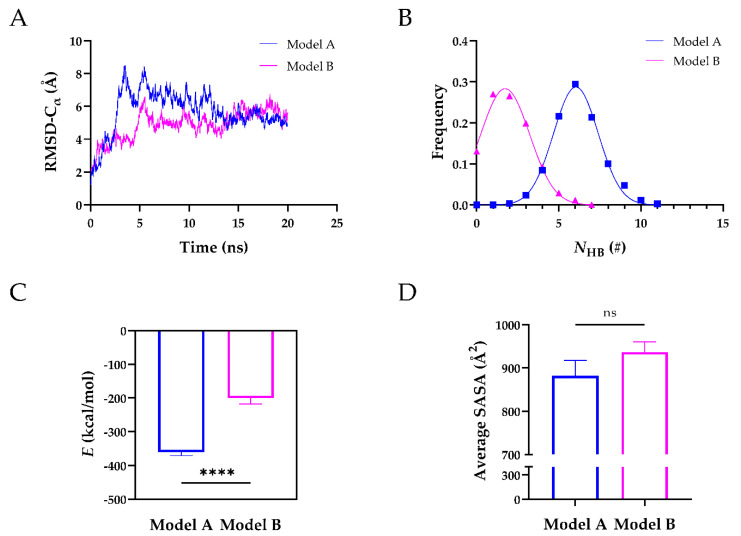
The structural stability of Model A was superior to that of Model B during the 20 ns equilibrium. (**A**) The time courses of Cα-RMSD for Model A and Model B; (**B**) the number of H-bonds (*N*_HB_) frequencies of the two models were fitted by Gaussian distribution; (**C**) plots of the mean interaction energy (*E*) of two models; (**D**) plots of the average SASA on the interface of two models. Data were presented as mean ± SEM. Differences on the interaction energy and average SASA of two models were analyzed with Student’s *t*-test (ns indicates not significant, **** indicates *p* < 0.0001, *n* = 20).

After obtaining their 3D structures, we ran 20 ns equilibrium simulations of Model A and Model B to further assess their structural stability. The time courses of the root-mean-square deviation (RMSD) of heavy atoms showed that both models reached their equilibrium state at 20 ns (Figure 2A). Further analysis revealed that the mean number of H-bonds (*N*_HB_) on the interface of Model A was ~6 with a Gaussian distribution, ~3-fold higher than that of Model B, suggesting that Model A was more stable than Model B (Figure 2B). The stability of Model A was also reflected by the interaction energy (Figure 2C). The interaction energy of Model A was −361.1 kcal/mol and ~2-fold lower than that of Model B (−200.7 kcal/mol). The average solvent accessible surface area (SASA) of Model A was comparable to that of Model B (Figure 2D). We also analyzed the H-bonds and salt bridges on the interface of the two Models (Table 2). During 20 ns equilibrium simulations, seven H-bonds and four salt bridges existed in Model A, whereas three H-bonds and one salt bridge existed in Model B. Four residues (Arg568, Arg660, Tyr661, and Tyr665) of exosite-3 formed H-bonds, and two of them (Arg568 and Arg660) formed salt bridges with the TSP8 domain in Model A. In comparison, only one residue (Arg568) of exosite-3 formed an H-bond with the TSP8 domain in Model B. These findings suggested that Model A was more stable, mediated by the interactions of exosite-3 and the TSP8 domain. Model A was thus selected for the subsequent simulations.

### 2.2. Arg1075, Asp1090, Arg1095, and Cys1130 Were the Key Residues on the TSP8 Domain Binding to the Spacer Domain

Simulations of Model A were performed for 40 ns equilibrium thrice to obtain three equilibrated conformations of the TSP8-Spacer complex (WT1, WT2, and WT3). The time courses of the heavy-atoms RMSD ascended rapidly, fluctuated, and finally reached a stable plateau after 30 ns around 4.0 Å, 5.0 Å, and 7.5 Å for WT1, WT2, and WT3 (Figure S1A), indicating that these three complexes have reached their equilibrated states. The *N*_HB_ across the interface of three complexes were fitted to the Gaussian distribution (Figure S1B–D), implying that the conformational space of each complex was complete.

**Figure 3:**
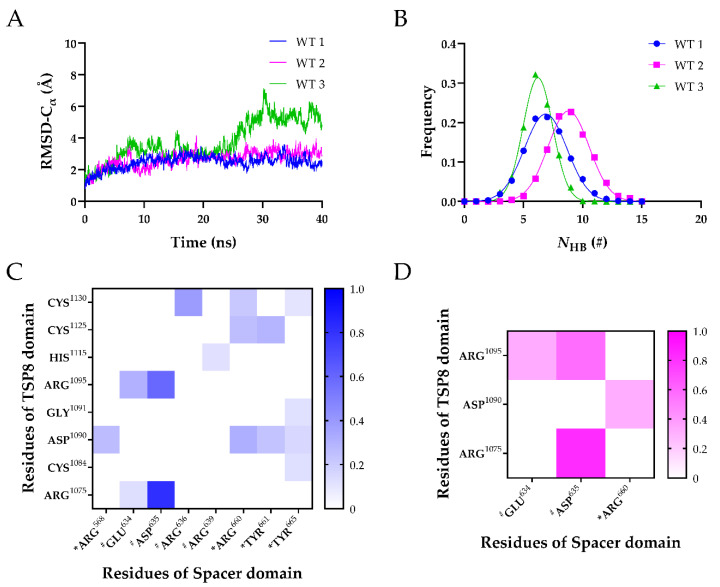
Three FMD simulations of the WT TSP8-Spacer complex. (**A**) Variation of Cα-RMSD of WT1 (blue), WT2 (magenta), and WT3 (green) with time. The Cα-RMSD value fluctuated mildly when the time exceeded 30 ns; (**B**) Gaussian fitting of the *N*_HB_ frequencies of three complexes (R^2^ > 0.99). The heat maps of average survival ratios of H-bonds (**C**) and salt bridges (**D**) on the interface of the TSP8-Spacer complex. * Indicates residues in exosite-3 of the Spacer domain, and # indicates residues in exosite-4 of the Spacer domain.

It was demonstrated that the key binding sites could be predicted via the stable H-bonds in free molecular dynamics (FMD) simulations [19]. Therefore, FMD simulations were performed for the equilibrated TSP8-Spacer complexes in parallel. As shown in Figure 3A, the time courses of Cα-RMSD of WT1 and WT2 were similar, with the Cα-RMSD climbing to ~3.0 Å within 10 ns and then fluctuating slightly. Large fluctuations of Cα-RMSD were observed after 20 ns in WT3, and the Cα-RMSD finally stabilized at ~5.5 Å. This indicated that WT1 and WT2 were more stable than WT3. The distributions of the *N*_HB_ on the interface of three complexes were fitted to a Gaussian distribution (Figure 3B), and the mean *N*_HB_ of WT1, WT2, and WT3 were 6.873 ± 1.797, 8.809 ± 1.736, and 6.218 ± 1.250, respectively. This implied that the interaction between the TSP8 domain and the Spacer domain was more stable in WT2. Therefore, we selected the structure of the last frame of WT2 during FMD stimulation for the subsequent mutations.

Considering that each FMD simulation obtained a possible conformational state of the TSP8-Spacer complex, we analyzed the H-bonds and salt bridges across the interface of all three complexes. Table 3 and Table 4 listed the H-bonds and salt bridges with an average survival ratio >10%. The H-bonds and salt bridges were classified into three categories: low (0~0.3), medium (0.3~0.55), and high (0.55~1.0) thermal stability according to their survival ratio value. The H-bonds with medium and high thermal stability were used to predict the key binding sites.

It was reported that exosite-3 was essential in mediating the auto-inhibition of ADAMTS13 [6,12,20]. Our MD simulations displayed that the exosite-3 residues did participate in the formation of H-bonds and salt bridges on the TSP8-Spacer complex, but their survival ratios were not high (Table 3 and Table 4). Among these H-bonds, Arg660-Asp1090 formed the fourth H-bond with moderate thermal stability (average survival ratio 0.313 ± 0.255). Except for that, all other H-bonds formed between the exosite-3 residues and the TSP8 domain exhibited low thermal stability. The average survival ratios of the H-bonds formed by Arg568, Tyr661, and Tyr665 with Asp1090 were 0.261 ± 0.109, 0.234 ± 0.191, and 0.155 ± 0.127, respectively. In addition to interacting with Asp1090, exosite-3 also formed H-bonds with other residues on the TSP8 domain. Arg660 formed the other two H-bonds with Cys1125 and Cys1130, and their average survival ratios were 0.251 ± 0.205 and 0.215 ± 0.176, respectively. The average survival ratio of the H-bond Tyr661-Cys1125 was 0.293 ± 0.178. Tyr665 formed three H-bonds with Cys1084, Gly1091, and Cys1130, but their average survival ratios were all below 0.15. Only one salt bridge was observed between the exosite-3 and the TSP8 domain. Arg660-Asp1090 formed the fourth salt bridge with a medium average survival ratio of 0.320 ± 0.261 (Table 4).

**Figure 4:**
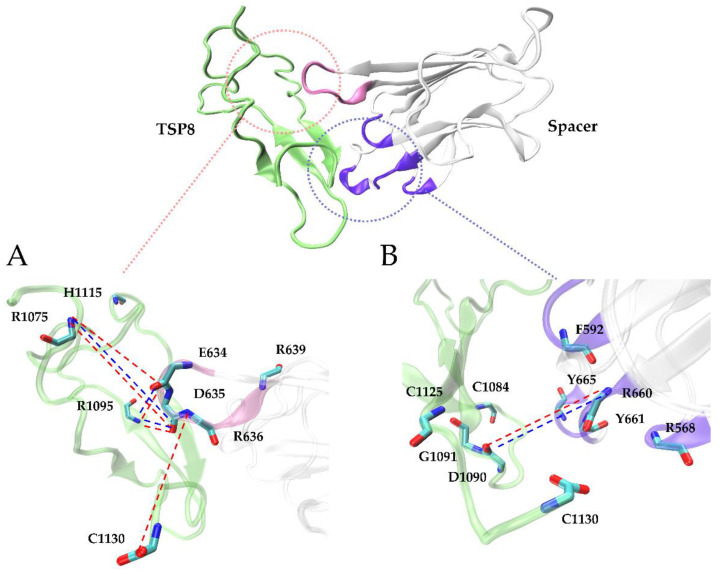
Cartoon representations of the 3D structure of WT TSP8-Spacer complex. Exosite-4 (pink) and exosite-3 (violet), both located at the distal end of the Spacer domain, are critical in stabilizing the complex. The detailed interactions between the TSP8 domain (green) and exosite-4 (inset A) or exosite-3 (inset B) are displayed here. We present all residues involved in these interactions, and the H-bonds (red dashed lines) and salt bridges (blue dashed lines) with an average survival ratio over 0.3.

Interestingly, Glu634, Asp635, and Arg636 on the Spacer domain formed H-bonds and salt bridges with residues on the TSP8 domain with high survival ratios. Glu634, Asp635, and Arg636 are the residues of exosite-4 that play important roles in the proteolysis of VWF73 and multimeric VWF [21]. Asp635 formed the top two H-bonds with Arg1075 (average survival ratio 0.815 ± 0.067) and Arg1095 (average survival ratio 0.591 ± 0.242). In addition, Asp635 also formed the top two salt bridges with Arg1075 (average survival ratio 0.829 ± 0.110) and Arg1095 (average survival ratio 0.569 ± 0.241). Arg636 formed the third H-bond with Cys1130 (average survival ratio 0.385 ± 0.149). Glu634 formed the fifth H-bond with Arg1095 and the 12th H-bond with Arg1075, with the average survival ratio of 0.305 ± 0.249 and 0.127 ± 0.068. Glu634 also formed the third salt bridge with Arg1095 (average survival ratio 0.330 ± 0.269). Apart from these three residues, Arg639 on exosite-4 formed a low survival ratio (0.117 ± 0.095) H-bond with His1115. The key interactions of the TSP8-Spacer complex are illustrated in Figure 4.

Arg1075, Asp1090, Arg1095, and Cys1130 on the TSP8 domain formed 11 out of 16 H-bonds and all four salt bridges with residues on the Spacer domain (Table 3 and Table 4). More importantly, they participated in the formation of the top five H-bonds with a high or medium thermal stability, suggesting they were the key residues mediating the TSP8-Spacer interactions. It is consistent with the previous report that point mutation in Arg1075 or Arg1095 was found in newborns with congenital TTP [22].

Taken together, both exosite-3 and exosite-4, particularly Glu634, Asp635, and Arg636 in the latter, were critical for the TSP8-Spacer interactions. Arg1075, Asp1090, Arg1095, and Cys1130 were predicted to be the key residues on the TSP8 domain.

### 2.3. GoF Mutations Attenuated the Binding of Exosite-3 but Enhanced the Binding of Exosite-4 to the TSP8 Domain

It was reported that after mutating the five key residues (R568K/F592Y/R660K/Y661F/Y665F) on the Spacer domain, the conformation of ADAMTS13 converted to an “open” state and its catalytic efficiency increased [5,12], indicating that these mutations weakened the binding of the Spacer domain to the C-terminal domains of ADAMTS13. To further validate our TSP8-Spacer model, the structure of the last frame of the FMD simulation of WT2 was selected and the five residues in the Spacer domain were mutated accordingly to obtain the GoF complex. The energy minimization and 40 ns equilibration were performed thrice, and three GoF complexes were obtained (GoF1, GoF2, and GoF3). The time courses of the Cα-RMSD of three complexes were similar, with high fluctuation during the first 30 ns and stabilization at ~6.0 Å for the last 10 ns, as shown in Figure S2A. The *N*_HB_ distributions of GoF1, GoF2, and GoF3 were fitted to the Gaussian distribution, with a mean *N*_HB_ of 6.856 ± 1.564, 6.099 ± 1.641, and 4.628 ± 1.336, respectively (Figure S2B–D).

**Figure 5:**
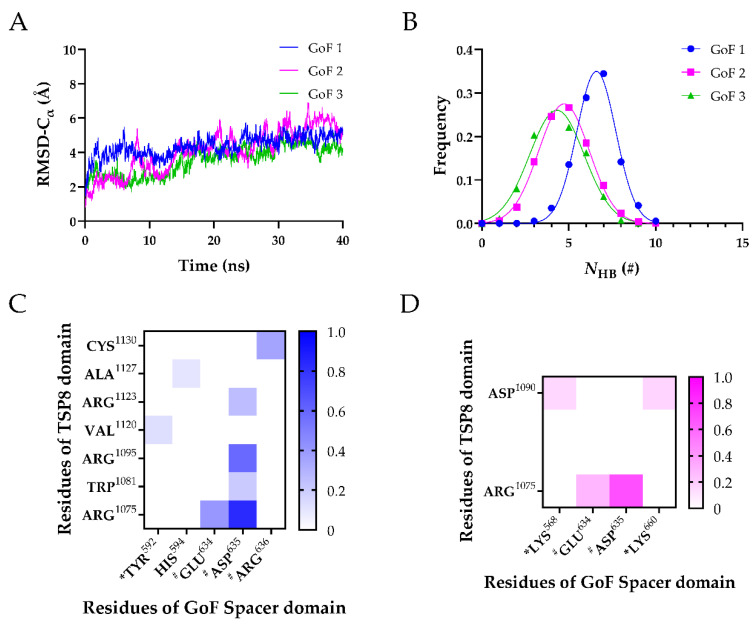
Three FMD simulations of the GoF complex. (**A**) Variation of Cα-RMSD with time for GoF1 (blue), GoF2 (magenta), and GoF3 (green). The Cα-RMSD fluctuated smoothly in a range of 1 Å when the time exceeded 30 ns; (**B**) Gaussian fitting of the *N*_HB_ frequencies of three GoF complexes (R^2^ > 0.98). The heat maps of the survival ratios of H-bonds (**C**) and (**D**) salt bridges on the interface of the GoF complex. * Indicates residues in exosite-3 of the Spacer domain, and # indicates residues in exosite-4 of the Spacer domain.

After obtaining their stable conformations, these GoF complexes were subject to FMD simulations. In Figure 5A, the Cα-RMSD of three GoF complexes climbed gradually. The mutations of the Spacer domain diminished the interactions on the interface by two aspects. First, the mean *N*_HB_ was reduced. The mean *N*_HB_ of the GoF complex was less than that of the WT complex. Additionally, the key binding sites on the Spacer domain that stabilized the complex were changed from two to one. Interactions of exosite-3 with the TSP8 domain were dramatically reduced. Only a newly formed H-bond (Tyr592-Val1120) and two weak salt bridges (Lys568-Asp1090 and Lys660-Asp1090) existed between the GoF exosite-3 and the TSP8 domain. Their average survival ratios were 0.129 ± 0.105 (Tyr592-Val1120), 0.156 ± 0.127 (Lys568-Asp1090), and 0.167 ± 0.136 (Lys660-Asp1090), respectively (Table 5 and Table 6). The stability of the complex was mainly dependent on exosite-4. Glu634, Asp635, and Arg636 on the Spacer domain formed six out of eight H-bonds and two out of four salt bridges with residues on the TSP8 domain (Table 5 and Table 6). Particularly, mutations on the Spacer domain increased the binding of Asp635 to the TSP8 domain. It bound four residues: Arg1075, Arg1095, Trp1081, and Arg1123. The H-bonds of Asp635-Arg1075 and Asp635-Arg1095 still ranked first and the second, with the corresponding average survival ratios of 0.835 ± 0.091 and 0.585 ± 0.239. The average survival ratios of two newly formed H-bonds by Asp635 were 0.206 ± 0.168 (with Trp1081) and 0.257 ± 0.210 (with Arg1123). In addition to Arg635, the H-bond formed by Glu634-Arg1075 (average survival ratio 0.406 ± 0.125) and Arg636-Cys1130 (average survival ratio 0.356 ± 0.259) were still essential for maintaining the bindings between the TSP8 domain and the GoF Spacer domain. These data demonstrated that the mutations of the Spacer domain attenuated the binding of exosite-3 but enhanced the binding of exosite-4 to the TSP8 domain.

### 2.4. The TSP8 Mutations Reduced the Stability of the Complex

To investigate whether the predicted residues (Arg1075, Asp1090, Arg1095, and Cys1130) on the TSP8 domain are critical, these four residues were all mutated to uncharged Ala to obtain the Mut complex. The Mut complex was subject to 40 ns equilibrium simulation thrice, and three Mut complexes (Mut1, Mut2, and Mut3) were obtained. The RMSD of heavy atoms for the Mut1, Mut2, and Mut3 fluctuated around 4.0 Å, 5.5 Å, and 5.0 Å, respectively (Figure S3A). The mean *N*_HB_ on the interface of each Mut complex was less than five during the 40 ns simulation (Figure S3B–D). Of note, the mean *N*_HB_ on the interface of Mut2 and Mut3 were as low as 2.439 ± 1.226 and 2.657 ± 2.159. These data suggested that mutations of the four residues on the TSP8 domain weakened the complex stability.

**Figure 6:**
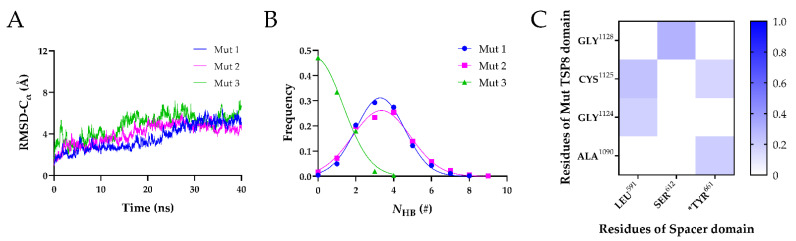
Three FMD simulations of the Mut complex. (**A**) Variation of Cα-RMSD with time for Mut1 (blue), Mut2 (magenta), and Mut3 (green). The Cα-RMSD fluctuated in a range of 1 Å when the time exceeded 20 ns. (**B**) Frequencies of the *N*_HB_ for three Mut complexes. The *N*_HB_ of the Mut1, Mut2 and Mut3 were fitted by Gaussian distribution (R^2^ > 0.98). (**C**) The heat maps of the average survival ratios of H-bonds on the interface of the Mut complex.

Subsequently, the equilibrated Mut complexes were subject to 40 ns FMD simulations. As shown in Figure 6A, the Cα-RMSD vs. time curves of three Mut complexes were similar, with the Cα-RMSD climbing to ~6 Å within 30 ns and then fluctuating slightly. The mean *N*_HB_ on the interface of these Mut complexes decreased dramatically (Figure 6B). The mean *N*_HB_ on the interface of the Mut1 and Mut2 was 3.293 ± 1.284 and 3.354 ± 1.535, respectively. In particular, almost no H-bonds were found on the interface of Mut3. We further analyzed the H-bonds and salt bridges on the interface of the complexes in detail. The four mutations resulted in the loss of all salt bridges on the interface of three Mut complexes. In addition, only five H-bonds were able to survive beyond 0.1 in three FMD simulations (Figure 6C and Table 7). The most stable H-bond was formed by Ser612-Gly1128, with an average survival ratio of 0.300 ± 0.212. The average survival ratios of the other four residue pairs were all below 0.3. We found that Tyr661 played a role in maintaining the stability of the Mut complex by interacting with Ala1090 (average survival ratio 0.194 ± 0.078) and Cys1125 (average survival ratio 0.164 ± 0.116). The average survival ratio of the H-bonds formed by Cys1125 and Gly1124 with Leu591 were 0.239 ± 0.169 and 0.182 ± 0.129, respectively.

The data above demonstrated that mutating Arg1075, Asp1090, Arg1095, and Cys1130 to Ala destabilized the TSP8-Spacer complex. This also emphasized the importance of these predicted residues on the TSP8 domain in the regulation of ADAMTS13 auto-inhibition.

**Figure 7:**
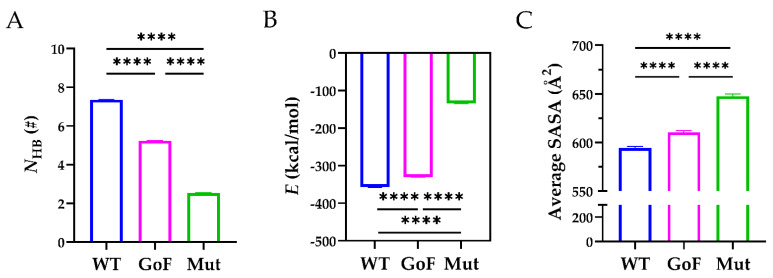
Comparisons of the WT, GoF, and Mut complexes. (**A**) The mean *N*_HB_, (**B**) the mean *E*, and (**C**) the average SASA across the interface of the WT (blue), GoF (magenta), and Mut (green) complexes. One-way ANOVA with a Bonferroni post hoc test was used to analyze the difference (**** indicates *p* < 0.0001, *n* = 3).

Next, we compared the stability of the WT, GoF, and Mut complexes. As shown in Figure 7A, the mean *N*_HB_ across the interface of the WT complex (7.130 ± 1.960) was significantly higher than those of the GoF complex (5.276 ± 1.803) and the Mut complex (2.064 ± 2.381). In addition, the mean *N*_HB_ of the GoF complex was significantly higher than that of the Mut complex. The corresponding interaction energy for the WT, GoF, and Mut complexes were −356.8 ± 1.143, −330.1 ± 0.889, and −134.1 ± 0.884 kcal/mol, and the differences among these three complexes were statistically significant (*p* < 0.0001) (Figure 7B). The interaction energy indicated that the WT complex was energy favorable compared to the other two complexes. Furthermore, the average SASA of the WT complex was 594.3 ± 1.870 Å^2^, significantly less than those of the GoF (610.5 ± 1.623 Å^2^) and Mut complex (647.6 ± 2.415 Å^2^), suggesting that mutations either in the Spacer or TSP8 domain partially exposed the hydrophobic binding core (Figure 7C). These data demonstrated that mutations on the Spacer or TSP8 domain impaired the Spacer-TSP8 binding, and the four specific mutations on the TSP8 domain resulted in the most unstable complex.

## 3. Discussion

Plasma exchange, the main therapy for acquired TTP (aTTP), still has a mortality rate of 10–20% in aTTP treatment [23]. The main reason for the high mortality rate is the rapid neutralization of ADAMTS13 by anti-ADAMTS13 IgGs produced by aTTP patients. Recombinant WT ADAMTS13 was shown to be safe and nonimmunogenic against congenital TTP (cTTP) in a phase I study [24]. However, it is still potentially neutralized by anti-ADAMTS13 IgGs in aTTP patient plasma. Better understanding the molecular basis of the structure of ADAMTS13 and selectively modifying the key residues might provide a new drug for the aTTP treatment. In line with this notion, it was suggested that GoF ADAMTS13 could be exploited for the development of a new drug as its efficient cleavage activity and its resistance to anti-ADAMTS13 auto-antibodies from patients with aTTP [12]. Recently, South et al. [25] identified a point mutation in Linker3 (located between TSP8 and CUB1 domains), Ala1144Val, which greatly enhanced the ADAMTS13 activity, effectively restored blood flow in a distal FeCl_3_ MCAo model, and reduced tissue hypoperfusion in a transient MCAo model of ischemia/reperfusion (I/R) injury. In the present study, we constructed the TSP8-Spacer complex by homology modelling and performed MD simulations. The key residues predicted in the present study, Arg1075, Asp1090, Arg1095, and Cys1130 on the TSP8 domain, might provide potential targets for new drug development.

It is well established that exosite-3 is crucial in mediating ADAMTS13 auto-inhibition [5,6,8]. GoF ADAMTS13 exhibits “open” conformation and increased proteolytic activity [12]. Recently, several studies have revealed that exosite-4 plays a key role in ADAMTS13 auto-inhibition as well. Kim and his colleagues demonstrated that exosite-3 directly interacted with Arg1326, Glu1387, and Glu1389 in CUB2, whereas exosite-4 bound to Trp1245, Trp1250, Lys1252, and Arg1272 in CUB1 [15]. Our previous study proposed that exosite-3 might bind to Glu1231, Asp1259, and Leu1251, whereas exosite-4 was capable of interacting with Arg1251 and Asp1261 in CUB1 [16]. The present results demonstrated that both exosite-3 and exosite-4 interact with the TSP8 domain, further emphasizing the significance of exosite-3 and exosite-4 in mediating ADAMTS13 auto-inhibition. Our MD simulations predicted that Arg1075, Asp1090, Arg1095, and Cys1130 on the TSP8 domain were key residues binding to the Spacer domain. In the WT complex, Asp1090 formed four H-bonds (with Arg568, Arg660, Tyr661, and Tyr665) and one salt bridge (with Arg660) (Table 3 and Table 4). In addition, the H-bond and salt bridge formed by Asp1090-Arg660 both exhibited medium thermal stability. Importantly, in the GoF and Mut complexes, the interactions of residue 1090 with exosite-3 were not completely abolished (Table 6 and Table 7). These data demonstrated that interactions between Asp1090 and exosite-3 might be the key to ensuring the TSP8-Spacer binding. In the WT complex, Arg1075 and Arg1095 tightly bound to exosite-4. Arg1075-Asp635 and Arg1095-Asp635 formed the top two H-bonds and top two salt bridges in the WT complex (Table 3 and Table 4), and these two residue pairs were less affected by the GoF mutations (Table 5 and Table 6). Cys1130 locating at the C-terminus of the TSP8 domain was less constrained and could contact both exosite-3 and exosite-4 in the WT complex. The GoF mutation ruined the H-bond formed by Cys1130 and exosite-3, but did not influence the H-bond formed by Cys1130 and exosite-4 (Cys1130-Arg636). Mutations of Arg1075, Asp1090, Arg1095, and Cys1130 into Ala resulted in the disappearance of all salt bridges on the interface (Table 7). These results indicate that Arg1075, Asp1090, Arg1095, and Cys1130 in the TSP8 domain are likely to be essential for ADAMTS13 to cleave VWF. Indeed, it was identified that causative mutations, i.e., p.R1075C, p.R1095W, and p.R1095Q, resulted in congenital ADAMTS13 deficiency [22].

In the present study, the GoF complex did not dissociate during 40 ns FMD, which is not consistent with previous reports. Under electron microscopy or small-angle X-ray scattering, the GoF ADAMTS13 adopts an “open” conformation [5,7]. The limited simulation time (40 ns) may be one of the reasons. This inconsistency may be also due to our mutation strategy. To investigate the interaction of the TSP8 domain and the GoF Spacer domain, we mutated the Spacer domain based on the stabilized WT TSP8-Spacer complex, resulting in a stable GoF mutation complex. Of note, our previous study with atomic force microscopy scanning demonstrated that 11.78% and 75.36% of GoF ADAMTS13 molecules in “closed” and intermediate states [26], implying that the interactions of the distal domains and the Spacer domain are not fully disrupted in some GoF ADAMTS13 molecules. Four key residues on the TSP8 domain are predicted in the present study. However, more experiments are needed to validate the significance of these residues.

Overall, we investigated the interaction of the TSP8 domain with the Spacer domain. MD simulations were used to depict the key residues on the TSP8 domain. The results revealed that Arg1075, Asp1090, Arg1095, and Cys1130 played important roles in maintaining the stability of the WT complex. Moreover, mutation of these four residues impaired the TSP8-Spacer binding, leading to an unstable complex.

## 4. Materials and Methods

### 4.1. Construction of the WT TSP8-Spacer Complex

The 3D structure of the Spacer domain (residues: Ser556-Pro682) was derived from the crystal structure of ADAMTS13-DTCS resolved by Akiyama et al. in 2009 (PDB ID: 3GHM; Resolution: 2.6 Å) [3]. As the crystal structure of the TSP8 domain is unavailable, the amino acid sequence of the TSP8 domain (residues: Cys1072 to Val1131) were uploaded to Phyre2 [27] to perform homology modeling. Among the 54 models of the TSP8 domain, we selected the top-ranked model, which shared the highest alignment coverage (92%) with the template thrombospondin-1 TSR domains 2 and 3 (PDB: 3R6B), to dock with the Spacer domain.

The SwarmDock Server was utilized to do the flexible docking [18], without the assumption of specific residues located on the interface of the Spacer or TSP8 domain. After getting all docking complexes, the top ten low energy models were investigated with VMD (Visual Molecular Dynamics). Two docking complexes, 67d and 47d, were selected based on the following two criteria: (1) partial or all five key residues of exosite-3 on the Spacer domain are on the interface; (2) Leu621, Glu622, and Asp623 on the loop β6–β7 of the Spacer domain are not on the interface, because they interact with the C_A_ region of the Cys domain, the adjacent N-terminal domain of the Spacer domain [3].

Subsequently, these two complexes were subject to the energy minimization and 20 ns equilibration simulation with NAMD (Nanoscale Molecular Dynamics) to analyze and select the most stable model for subsequent experiments. The optimal TSP8-Spacer docking complex was then subjected to energy minimization, equilibrium, and FMD simulations thrice to re-analyze the stability of the WT TSP8-Spacer complex [19].

### 4.2. Construction of the Mutated TSP8-Spacer Complexes

Based on the FMD simulation of the WT TSP8-Spacer complex, we mutated the predicted key residues on the TSP8 domain to Ala to obtain the TSP8-mutated complex (named Mut complex). We also mutated exosite-3 (R568K/F592Y/R660K/Y661F/Y665F) of the Spacer domain to obtain the GoF Spacer-TSP8 complex (named GoF complex). These two complexes were then respectively subjected to three independent energy minimization, equilibrium, and FMD simulations to further explore the effects of these mutations on the interaction of the Spacer domain and the TSP8 domain.

### 4.3. Molecular Dynamics Simulation

By utilizing VMD, the WT, GoF, and Mut complexes were immersed into a rectangular box composed of TIP3P water molecules. Protein atoms were 25 Å away from the wall. Na^+^ and Cl^−^ were added to the system at a concentration of 150 mM to mimic the physiological environment. Particle mesh Ewald (PME) [28] was used to calculate the electrostatic interactions, and a 12 Å switching cut-off was set for electrostatic and van der Waals interaction.

**Figure 8:**
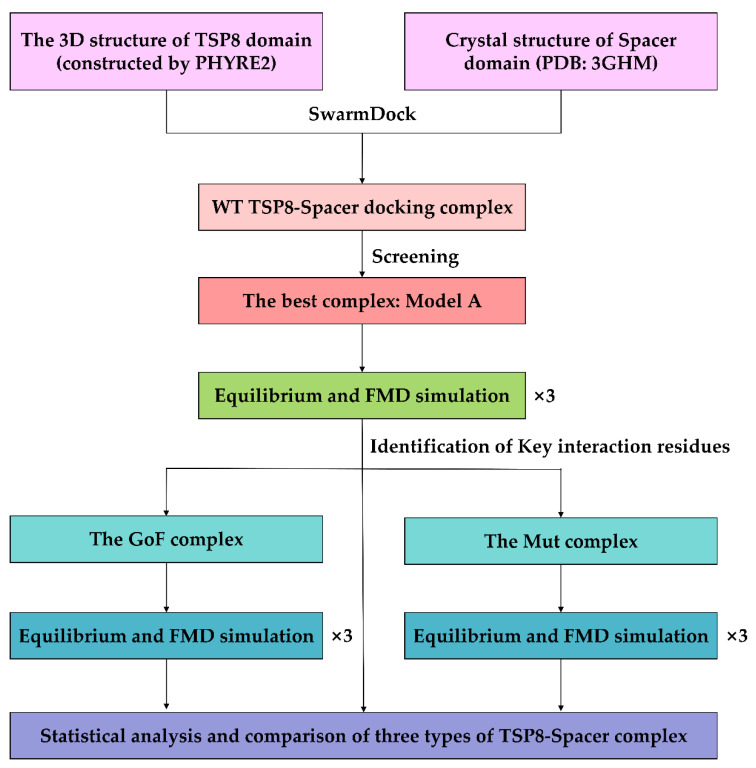
Workflow diagram of the simulation process.

The system was subjected to the energy minimization in the CHARMM27 all-atom force field [29] with NAMD. The energy minimization can be divided into three parts: (1) fixed all atoms in a complex; (2) fixed the backbone of a complex and other atoms were free; and (3) all atoms were free. After running the energy minimization for 15,000 steps in each part thrice (the time step of 2 fs), the system was subject to 40 ns equilibration at the constant temperature of 310 K and the constant pressure of 1 atm. RMSD of heavy atoms and the time course of the *N*_HB_ on the interface were analyzed to determine whether the system had been equilibrated. When three different equilibrated poses for a complex were obtained, they were used as the initial conformation for the following FMD simulations in parallel. The FMD simulation was performed for 40 ns without controlling the temperature and pressure, which is similar to the NVE ensemble [30]. Figure 8 showed the overall workflow of the complex construction and simulation.

### 4.4. Data Analysis

#### 4.4.1. The Selection of Key Residues

An H-bond was defined as a bond with an angle greater than 150° and a distance less than 3.5 Å between the donor and acceptor atoms. A salt bridge was defined as a bond formed by -O on the side chain of acidic amino acid and -N on the side chain of basic amino acid, with the bond length less than 4 Å. The survival ratio of an H-bond or a salt bridge was defined as the bond dwell time over the simulation duration, which was used to characterize the thermal stability of a complex. We used heat maps to illustrate H-bonds and salt bridges with an average survival ratio greater than 10%. The key residues were defined if those residues formed H-bonds or salt bridges with an average survival ratio greater than 30%.

#### 4.4.2. Structural Stability Analysis

We used VMD tools to analyze the trajectories of all simulations. Cα-RMSD was applied to compute the deviation of a conformation from the initial state and describe the conformational stability [31]. The SASA characterized the degree of the hydrophobic core exposure with a 1.4 Å cutoff [32]. To estimate the binding energy of a complex, we calculated the interaction energy (*E*) of a complex with a combination of the van der Waals energy and electrostatic energy.

#### 4.4.3. Statistical Analysis

The *N*_HB_ was fitted with Gaussian distribution. For a two-group comparison, the unpaired Student’s *t*-test (parametric) was used. For a multiple-group comparison, one-way ANOVA was used with a Bonferroni post hoc test. The value of *p* < 0.05 was considered statistically significant. GraphPad Prism 8.0.1 software was used to analyze the data.

## Figures and Tables

**Table 1:** Residue interactions on the static interface of Model A and B.

Model	H-Bond		Salt Bridge	
	TSP8	Spacer	TSP8	Spacer
Model A	VAL^1078^	GLU^634^	ARG^1075^	GLU^634^
	GLY^1089^	*ARG^568^	ASP^1090^	*ARG^568^
	ASP^1090^	*ARG^660^	ARG^1095^	ASP^635^
	ARG^1094^	*TYR^665^	-	-
	ARG^1095^	ASP^635^	-	-
	LYS^1118^	HIS^588^	-	-
	ARG^1123^	ARG^636^	-	-
Model B	CYS^1072^	*ARG^568^	-	-
	TYR^1074^	*ARG^660^	-	-
	GLN^1103^	ARG^636^	-	-
	ALA^1104^	*PHE^592^	-	-
	PRO^1109^	*ARG^660^	-	-

* Indicates residues in exosite-3 of the Spacer domain.

**Table 2:** Residue interactions on the interface of Model A and Model B at equilibrium.

Model	TSP8	Spacer	Survival Ratio	
			H-Bond	Salt Bridge
Model A	ARG^1095^	ASP^635^	0.971	0.988
	ASP^1090^	*TYR^661^	0.228	-
	ASP^1090^	*ARG^660^	0.637	0.587
	ASP^1090^	*ARG^568^	0.587	0.381
	LYS^1118^	ASP^672^	0.180	-
	SER^1085^	*TYR^665^	0.165	-
	ARG^1075^	GLU^634^	0.808	0.706
Model B	ASP^1111^	*ARG^568^	0.212	-
	THR^1098^	ARG^636^	0.188	-
	ARG^1096^	ASP^635^	0.508	0.218

* Indicates residues in exosite-3 of the Spacer domain.

**Table 3:** The H-bonds on the interface of the WT TSP8-Spacer complex with an average survival ratio >10%.

Ranking	TSP8	Spacer	Survival Ratio			Average
			WT 1	WT 2	WT 3	(Mean ± SEM)
1	ARG^1075^	^#^ASP^635^	0.652	0.911	0.883	0.815 ± 0.067
2	ARG^1095^	^#^ASP^635^	0.843	0.930	0.000	0.591 ± 0.242
3	CYS^1130^	^#^ARG^636^	0.051	0.423	0.681	0.385 ± 0.149
4	ASP^1090^	*ARG^660^	0.000	0.938	0.000	0.313 ± 0.255
5	ARG^1095^	^#^GLU^634^	0.000	0.000	0.915	0.305 ± 0.249
6	CYS^1125^	*TYR^661^	0.718	0.161	0.000	0.293 ± 0.178
7	ASP^1090^	*ARG^568^	0.446	0.336	0.000	0.261 ± 0.109
8	CYS^1125^	*ARG^660^	0.000	0.754	0.000	0.251 ± 0.205
9	ASP^1090^	*TYR^661^	0.000	0.703	0.000	0.234 ± 0.191
10	CYS^1130^	*ARG^660^	0.000	0.000	0.646	0.215 ± 0.176
11	ASP^1090^	*TYR^665^	0.466	0.000	0.000	0.155 ± 0.127
12	ARG^1075^	^#^GLU^634^	0.285	0.097	0.000	0.127 ± 0.068
13	CYS^1084^	*TYR^665^	0.365	0.000	0.000	0.122 ± 0.099
14	GLY^1091^	*TYR^665^	0.353	0.000	0.000	0.118 ± 0.096
15	HIS^1115^	^#^ARG^639^	0.350	0.000	0.000	0.117 ± 0.095
16	CYS^1130^	*TYR^665^	0.000	0.314	0.000	0.105 ± 0.085

* Indicates residues in exosite-3 of the Spacer domain, and # indicates residues in exosite-4 of the Spacer domain.

**Table 4:** The salt bridges on the interface of the WT TSP8-Spacer complex with an average survival ratio >10%.

Ranking	TSP8	Spacer	Survival Ratio			Average
			WT 1	WT 2	WT 3	(Mean ± SEM)
1	ARG^1075^	^#^ASP^635^	0.560	0.985	0.943	0.829 ± 0.110
2	ARG^1095^	^#^ASP^635^	0.716	0.993	0.000	0.569 ± 0.241
3	ARG^1095^	^#^GLU^634^	0.000	0.000	0.989	0.330 ± 0.269
4	ASP^1090^	*ARG^660^	0.000	0.960	0.000	0.320 ± 0.261

* Indicates residues in exosite-3 of the Spacer domain, and # indicates residues in exosite-4 of the Spacer domain.

**Table 5:** The H-bonds on the interface of the GoF complex with an average survival ratio >10%.

Ranking	TSP8	Spacer	Survival Ratio			Average
			GoF 1	GoF 2	GoF 3	(Mean ± SEM)
1	ARG^1075^	^#^ASP^635^	0.992	0.893	0.621	0.835 ± 0.091
2	ARG^1095^	^#^ASP^635^	0.844	0.000	0.910	0.585 ± 0.239
3	ARG^1075^	^#^GLU^634^	0.668	0.414	0.137	0.406 ± 0.125
4	CYS^1130^	^#^ARG^636^	0.987	0.079	0.000	0.356 ± 0.259
5	ARG^1123^	^#^ASP^635^	0.000	0.000	0.771	0.257 ± 0.210
6	TRP^1081^	^#^ASP^635^	0.618	0.000	0.000	0.206 ± 0.168
7	VAL^1120^	*TYR^592^	0.000	0.000	0.386	0.129 ± 0.105
8	ALA^1127^	HSD^594^	0.000	0.304	0.000	0.101 ± 0.083

* Indicates residues in exosite-3 of the Spacer domain, and # indicates residues in exosite-4 of the Spacer domain.

**Table 6:** The salt bridges on the interface of the GoF complex with an average survival ratio >10%.

Ranking	TSP8	Spacer	Survival Ratio			Average
			GoF 1	GoF 2	GoF 3	(Mean ± SEM)
1	ARG^1075^	^#^ASP^635^	0.666	0.950	0.450	0.689 ± 0.118
2	ARG^1075^	^#^GLU^634^	0.000	0.000	0.858	0.286 ± 0.233
3	ASP^1090^	*LYS^660^	0.000	0.500	0.000	0.167 ± 0.136
4	ASP^1090^	*LYS^568^	0.000	0.467	0.000	0.156 ± 0.127

* Indicates residues in exosite-3 of the Spacer domain, and # indicates residues in exosite-4 of the Spacer domain.

**Table 7:** The H-bonds on the interface of the Mut complex with an average survival ratio >10%.

Ranking	TSP8	Spacer	Survival Ratio			Average
			Mut 1	Mut 2	Mut 3	(Mean ± SEM)
1	GLY^1128^	SER^612^	0.900	0.000	0.000	0.300 ± 0.212
2	CYS^1125^	LEU^591^	0.000	0.718	0.000	0.239 ± 0.169
3	ALA^1090^	*TYR^661^	0.101	0.413	0.068	0.194 ± 0.078
4	GLY^1124^	LEU^591^	0.546	0.000	0.000	0.182 ± 0.129
5	CYS^1125^	*TYR^661^	0.492	0.000	0.000	0.164 ± 0.116

* Indicates residues in exosite-3 of the Spacer domain.

## Data Availability

The data is available on request from the author.

## Supplementary Materials

The following are available online. Figure S1: Three equilibrium simulations of the WT complex. Figure S2: Three equilibrium simulations of the GoF complex. Figure S3: Three equilibrium simulations of the Mut complex.

## Sample Availability

Samples of the compounds are not available from the authors.
