# Supplementary material for: Residues R1075, D1090, R1095, and C1130 Are Critical in ADAMTS13 TSP8-Spacer Interaction Predicted by Molecular Dynamics Simulation
Source: Molecules. 2021 Dec 12;26(24):7525. doi: 10.3390/molecules26247525 (PMC8703438; doi:10.3390/molecules26247525)
Supplement: Supplementary file 1 [file molecules-26-07525-s001.zip › molecules-1490558-supplementary.pdf]

molecules

MDPI

Supplementary Materials

Residues R1075, D1090, R1095, and C1130 Are Critical in ADAMTS13 TSP8-Spacer Interaction Predicted by Molecular Dynamics Simulation

Zhiwei Wu [1,2], Junxian Yang [1,2], Xubin Xie [1], Guangjian Liu [2], Ying Fang [1], Jianhua Wu [1] and Jiangguo Lin [2,3,*]

[1] Institute of Biomechanics/School of Biology and Biological Engineering, South China University of Technology, Guangzhou 510006, China.
[2] Research Department of Medical Sciences, Guangdong Provincial People's Hospital, Guangdong Academy of Medical Sciences, Guangzhou 510080, China.
[3] Department of Emergency Medicine, Guangdong Provincial People's Hospital, Guangdong Academy of Medical Sciences, Guangzhou 510080, China.
* Correspondence: linjiangguo@gdph.org.cn

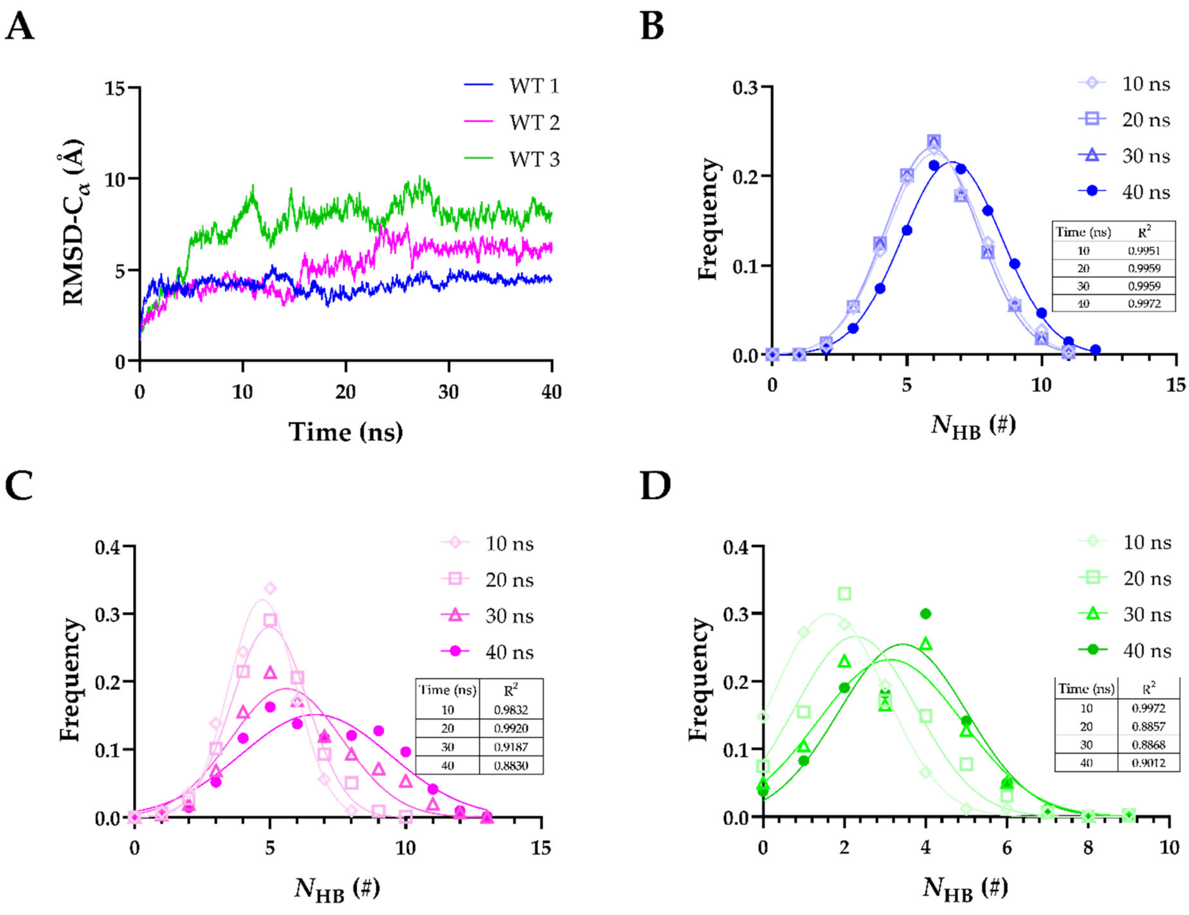

Figure S1. Three equilibrium simulations of the WT TSP8-Spacer complex. (**A**) Variation of Cα-RMSD of WT1 (blue), WT2 (magenta), and WT3 (green) with time. The Cα-RMSD fluctuated in a range of 1 Å when the time exceeded 30 ns. (**B–D**) Distributions of N_{HB} during 40 ns equilibrium. The N_{HB} from equilibrium simulation times of 10, 20, 30, and 40 ns were fitted to the Gaussian distribution (solid line with various shades, blue: WT1, magenta: WT2, and green: WT3). The R^2 values for these three complexes of 40 ns equilibrium simulations were 0.9972, 0.8830, and 0.9012, suggesting that the conformation space of the complex sampled in equilibrium is quasi-complete.

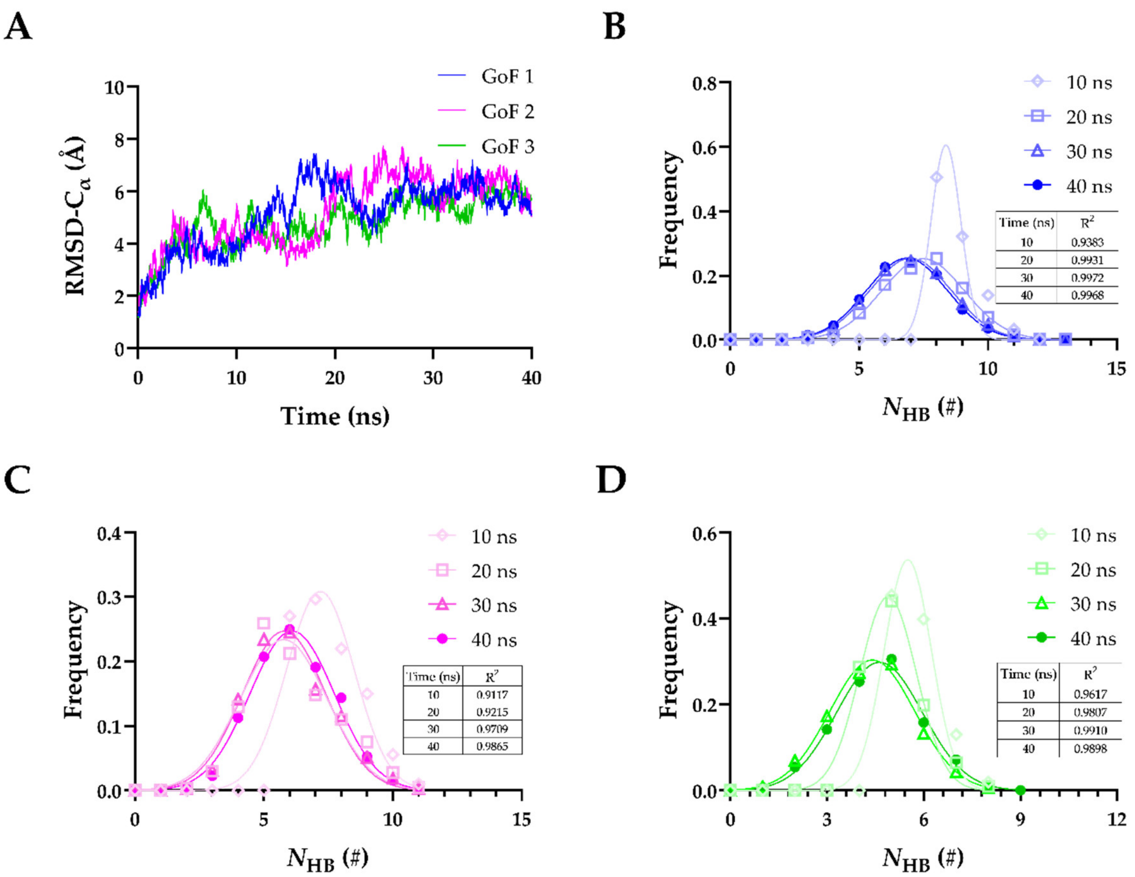

Figure S2. Three equilibrium simulations of the GoF complex. (**A**) Variation of Cα-RMSD of the GoF1 (blue), GoF2 (magenta), and GoF3 (green) with time. The Cα-RMSD fluctuated in a range of 1 Å when the time exceeded 30 ns. (**B**–**D**) Distributions of N_{HB} during 40 ns equilibrium. The N_{HB} from equilibrium simulation times of 10, 20, 30, and 40 ns were fitted to the Gaussian distribution (solid line with various shades, blue: GoF1, magenta: GoF2, and green: GoF3). The R^2 values for these three simulations of 40 ns equilibrium simulations were 0.9968, 0.9865, and 0.9898, suggesting that the conformation space of the complex sampled in equilibrium is quasi-complete.

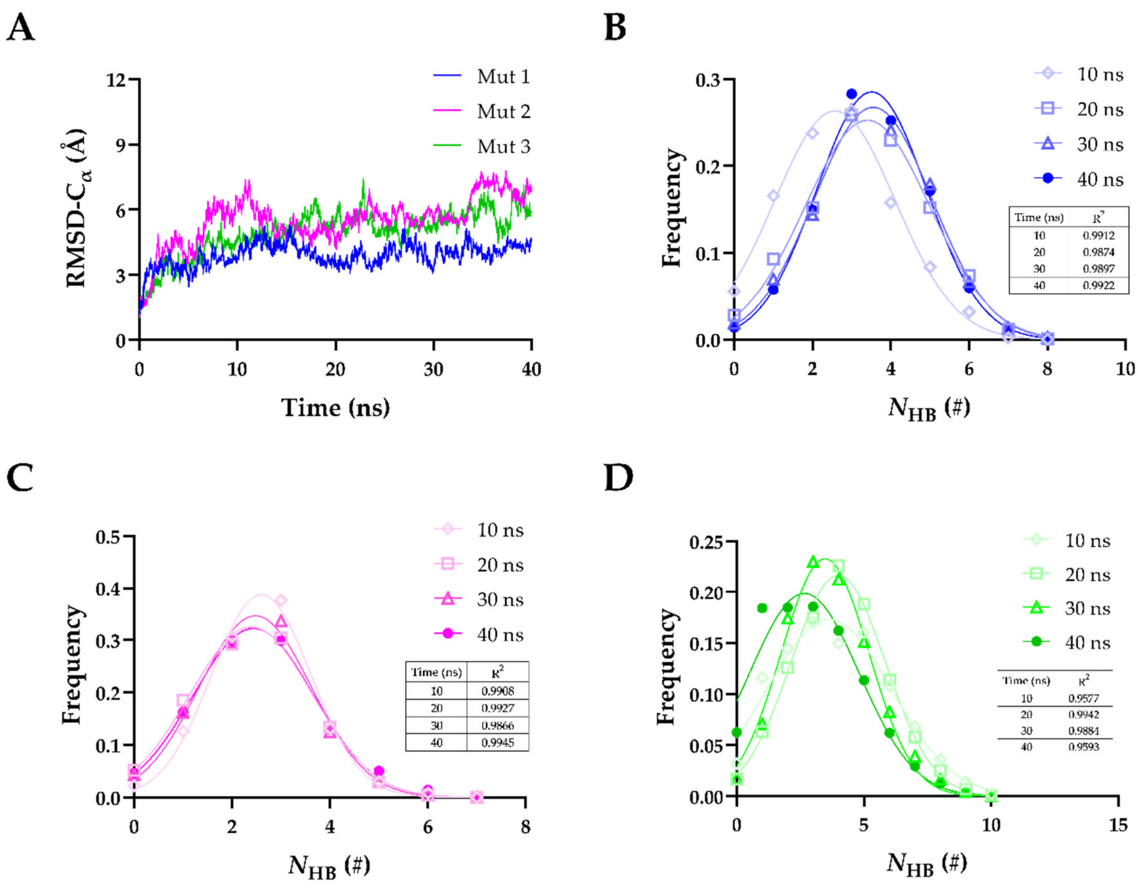

Figure S3. Three equilibrium simulations of the Mut complex. (**A**) Variation of Cα-atoms RMSD of the Mut1 (blue), Mut2 (magenta), and Mut3 (green) with time. The Cα-RMSD fluctuated slightly when the time exceeded 10 ns. (**B**–**D**) Distributions of N_{HB} during 40 ns equilibrium. The N_{HB} from equilibrium simulation times of 10, 20, 30, and 40 ns were fitted to the Gaussian distribution (solid line with various shades, blue: Mut1, magenta: Mut2, and green: Mut3). The R^2 values for these three simulations of 40 ns equilibrium simulations were 0.9922, 0.9945, and 0.9593, suggesting that the conformation space of the complex sampled in equilibrium is quasi-complete.
